# Cyanide produced with ethylene by ACS and its incomplete detoxification by β-CAS in mango inflorescence leads to malformation

**DOI:** 10.1038/s41598-019-54787-7

**Published:** 2019-12-04

**Authors:** Mohammad Wahid Ansari, Shail Kaushik, Gurdeep Bains, Suresh Tula, Bhavana Joshi, Varsha Rani, Ratnum Kaul Wattal, Randeep Rakwal, Alok Shukla, Ramesh Chandra Pant, Renu Tuteja, Narendra Tuteja

**Affiliations:** 1 International Centre for Genetic Engineering and Biotechnology (ICGEB), Aruna Asaf Ali Marg, 110067 New Delhi, India; 2 Department of Plant Physiology, CBSH, G. B. Pant University of Agriculture and Technology, Pantnagar, 263145 India; 3 Institute of Plant Genetics and Crop Plant Research (IPK), Molecular Plant Nutrition, Corrensstr. 3, 06466 Gatersleben, Germany; 4 Department of Botany, Zakir Husain Delhi College (University of Delhi), Jawahar Lal Nehru Marg, 110002 New Delhi, India; 5 Faculty of Health and Sport Sciences, University of Tsukuba, 1-1-1 Tennodai, Tsukuba, 305-8574 Ibaraki Japan

**Keywords:** Plant sciences, Biochemistry, Transcriptomics

## Abstract

Malformation of mango inflorescences (MMI) disease causes severe economic losses worldwide. Present research investigates the underlying causes of MMI. Results revealed significantly higher levels of cyanide, a by-product of ethylene biosynthesis, in malformed inflorescences (MI) of mango cultivars. There was a significant rise in *ACS* transcripts, ACS enzyme activity and cyanide and ethylene levels in MI as compared to healthy inflorescences (HI). Significant differences in levels of methionine, phosphate, S-adenosyl-L-methionine, S-adenosyl-L-homocysteine, ascorbate and glutathione, and activities of dehydroascorbate reductase and glutathione reductase were seen in MI over HI. Further, a lower expression of β-cyanoalanine synthase (*β-CAS*) transcript was associated with decreased cellular β-CAS activity in MI, indicating accumulation of unmetabolized cyanide. TEM studies showed increased gum-resinosis and necrotic cell organelles, which might be attributed to unmetabolized cyanide. In field trials, increased malformed-necrotic-inflorescence (MNI) by spraying ethrel and decreased MNI by treating with ethylene inhibitors (silver and cobalt ions) further confirmed the involvement of cyanide in MMI. Implying a role for cyanide in MMI at the physiological and molecular level, this study will contribute to better understanding of the etiology of mango inflorescence malformation, and also help manipulate mango varieties genetically for resistance to malformation.

## Introduction

Mango inflorescence is a branched raceme with pedicellate flowers arranged acropetally, which bears 500 yellowish-green bisexual and male flowers in variable proportion^1,2^. However, malformed inflorescences do not set fruit; therefore, inflorescence malformation in mango trees is a terrible disease^3^ for mango farmers in that it reduces fruit productivity by 50–80 percent each year^4^. The disease was first identified in India by Marries, an expert mango grower from the Darbhanga district of Bihar^5,6^. Since then, it has been recognized in other mango producing countries such as Pakistan, the Middle East, Egypt, South Africa, Brazil, Sudan, Central America, Mexico, Cuba, Malaysia, Australia, Israel, UAE, Bangladesh, Sultanate of Oman, Southern Spain, China, United States and Senegal^6^. Mango malformation is a complex and destructive disease, which is exacerbated by the inadequate knowledge of its etiology.

Previously, we have reported higher content of ethylene in MMI^7,8^. In addition to producing ethylene^9^, *Fusarium mangiferae* leads to the overexpression of key mango genes^10,11^, which affects plant hormone homeostasis^12^ and results in stress hormone ethylene formation^7^. Cyanide, a co-product of ethylene synthesis exerts adverse, toxic and long-term effects on plant growth and development^13,14^. Therefore, cyanide derived from ethylene could contribute to the development of malformation. In non-cyanogenic plants, cyanide is usually not detoxified by the β-CAS enzyme under adverse condition^13^. Thus, the accumulated unmetabolized cyanide causes abnormal development of flower via restricted cell elongation, reduced elongation of rachis, deformation of reproductive organs, trichome formation, necrosis and cell death^13,15,16^. The expression analysis of key genes of cyanide and ethylene metabolism during the course of malformation is decisive to determine the etiology of disease, and, which has not been studied so far.

Cyanide is produced during the ethylene biosynthesis pathway in which the enzyme ACC synthase (ACS) catalyzes the rate-limiting step^17,18^; further, increased endogenous cyanide is associated with enhanced cellular ACS activity^19^. Sato *et al*.^20^ cloned the first *ACS* gene from *Cucurbita pepo*. *ACS* gene has also been characterized from many other plant species^21,22^. Nevertheless, *ACS* genes in the mango malformation system have not yet been investigated. In the methionine cycle, cyanide synthesis along with ethylene takes place without demanding additional methionine^23,24^. This will cause accumulation of inorganic phosphorous (PPi and Pi)^25^. The last step of cyanide synthesis is during the conversion of ACC to ethylene catalyzed by ACC oxidase (ACO), which is oxygen dependent, and utilizes Fe^2+^ and ascorbate (ASA)^26^. Dehydroascorbate reductase (DHAR) enzyme reduces dehydroascorbate (DHA) to ASA using glutathione (GSH) as an electron donor^27^. DHAR, GSH, and GR maintain the endogenous ASA pool^28^. The cyanide is a degraded product of ACC, which is derived from methionine. The methionine cycle revolving at a higher pace explains the higher content of cyanide affecting respiratory rate and flower growth^29^. Additionally, besides cyanide the level of other byproducts of ethylene biosynthesis such as ascorbate, inorganic phosphate, and methionine and other biomolecules and antioxidants might be crucial for the development of malformation, and which also needs to be examined. Cyanide plays a dual role in plants; it may be toxic at high concentration or may have regulatory role towards stress response^30^. β-cyanoalanine synthase (β-CAS) is primarily responsible for cyanide detoxification in plants^31^. Most plants were reported to exhibit higher cyanide levels together with low level of β-CAS in response to stress^29,32^. β-CAS enzyme is localized to mitochondria^33^ to protect the electron transport chain of mitochondria at the site of cyanide production^34^. Although *β-CAS* genes were studied in several plant species^35^, there are no reports of *β-CAS* transcript accumulation and cyanide detoxification in the mango malformation system.

The present study aims to study the comparative expression profiles of *ACS* and *β-CAS* in malformed and healthy inflorescence (abbreviated as, MI and HI, respectively) of three mango cultivars – Mallika (Mk), Ramkela (Rk), and Langra (Ln) differing in their degree of susceptibility to mango malformation disease. We have used these cultivars to correlate physiological and molecular study with cultivar susceptiblity. We investigated the endogenous cyanide content, ethylene pool, and levels of other biomolecules, which may trigger the malformed necrotic inflorescence. Further, transmission electron microscopy was utilized to study the morphological differences in malformed and healthy sections of the mango cultivar Rk. Finally, the response of exogenously applied inhibitors of cyanide/ethylene in the incidence of mango malformation under field conditions was also examined. Our study attempts to provide insights into the etiology of mango malformation and to help devise strategies to control the malformation of mango inflorescences (MMI) disease.

## Results

### Expression analysis of ACS transcript, and measuring the ACS activity, ethylene content and cyanide level

**Figure 1:**
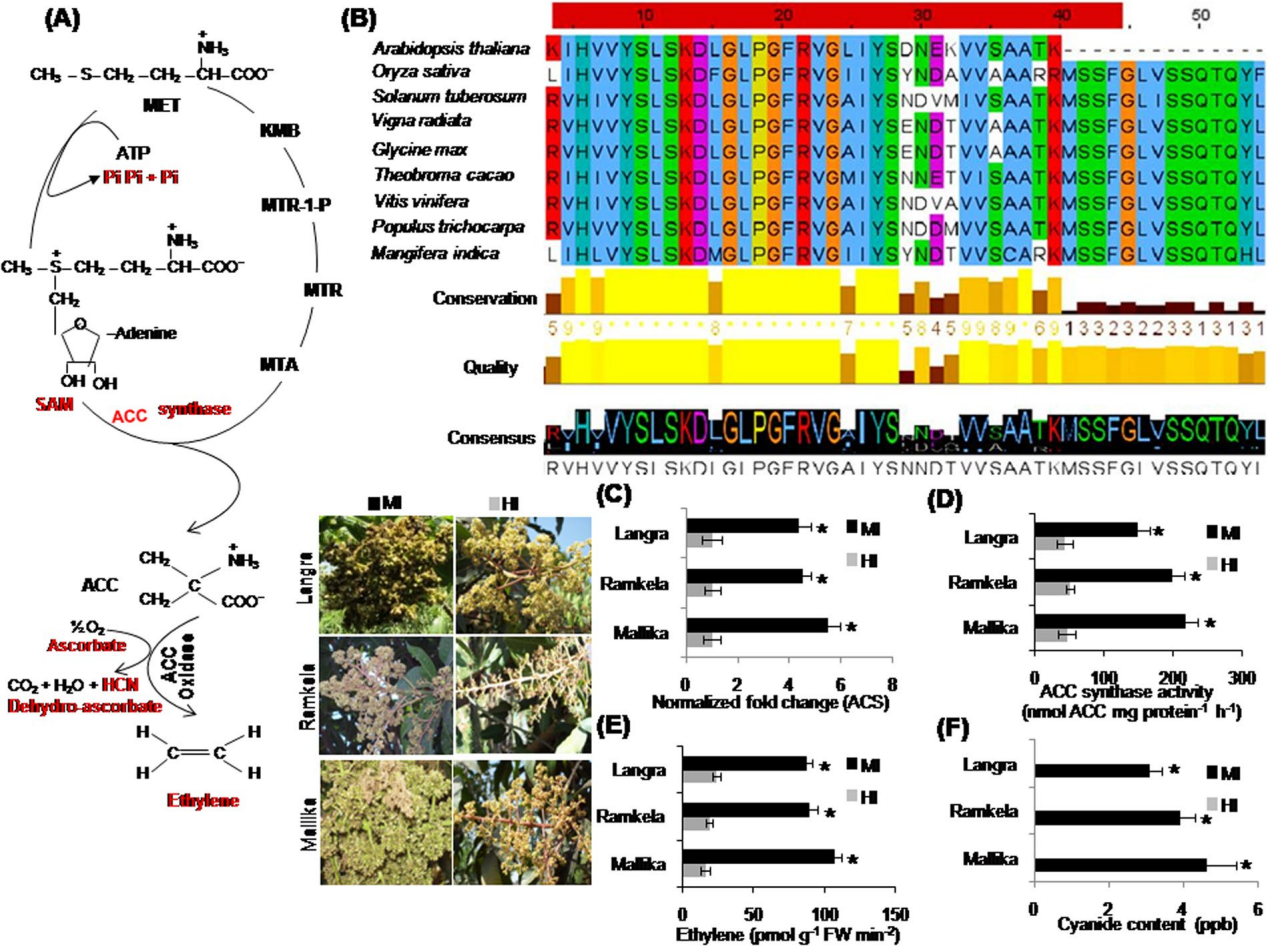
Ethylene biosynthesis, amino acid sequence alignment for 1-aminocyclopropane-1-carboxylic acid (*ACS*), and endogenous *ACS* transcript level, ACS enzyme activity and ethylene content in mango inflorescence. Ethylene biosynthesis in mango and regulatory ACC synthase enzyme and byproducts (ascorbate, inorganic phosphate, methionine and cyanide). (**A**) The amino acid sequence alignment of *ACS* gene of plant species. s(**B**) The endogenous transcript levels of *ACS*. (**C**) ACC synthase enzyme activity (nmol ACC mg protein^−1^ h^−1^) (**D**) and ethylene content (pmol g^−1^ FW min^−2^) (**E**) in the malformed (MI) and healthy inflorescence (HI) of mango cultivars (Mk, Rk and Ln). Cyanide content (ppb) in MI (**F**) of mango cultivars while cyanide content in HI was at undetectable levels. The MI and HI of Mk, Rk and Ln are shown on the left side of the bar diagrams. Data represent means ± SE of at least 3 replicates. ‘*’Indicates a significant difference between the malformed and healthy inflorescence at the 0.05 level of significance.

Ethylene biosynthesis is conserved in mango plants^7^. Its main regulatory enzymes are ACC synthase and ACC oxidase which are positively or negatively altered by abiotic stresses^17^. The byproducts of ethylene biosynthesis in plants are methionine, S-adenosyl-L-methionine (SAM), inorganic phosphate, ascorbate and cyanide (Fig. 1A). The amino acid sequence alignment of ACS of diverse plant species shows apparent regions of conservation, quality, and consensus between 4–44 amino acids (Fig. 1B). The MI showed a significant increase in the transcript levels of *ACS* in all tested cultivars as compared with HI. The transcript levels of *ACS* in the MI of Mk, Rk and Ln cultivars were 5.46, 4.5, and 4.33 fold higher than in the HI (Fig. 1C). The ACS activity (nmol ACC mg protein^−1^ h^−1^) was the highest in the MI of the susceptible mango cultivar Mk (217) followed by relatively tolerant cultivars such as Rk and Ln (199 and 148) as compared with the HI (Fig. 1D). The endogenous ethylene content (pmol g^−1^ FW min^−2^) of floral tissues was significantly elevated in MI as compared to HI (Fig. 1E). The cultivar Mk showed the highest (107.19), and Ln showed the lowest endogenous ethylene (87.21) content (Fig. 1E). The endogenous cyanide level (ppb) of floral tissues was significantly increased in MI as compared to HI (Fig. 1F). The cultivar Mk revealed the maximum (4.6), and Ln exhibited the lowest endogenous cyanide (3.1) level (Fig. 1F).

### Endogenous methionine content, the cations and anions concentration, and S-adenosyl-L-methionine (SAM) and S-adenosyl-L-homocysteine (SAH) content and the SAH/SAM ratio

**Figure 2:**
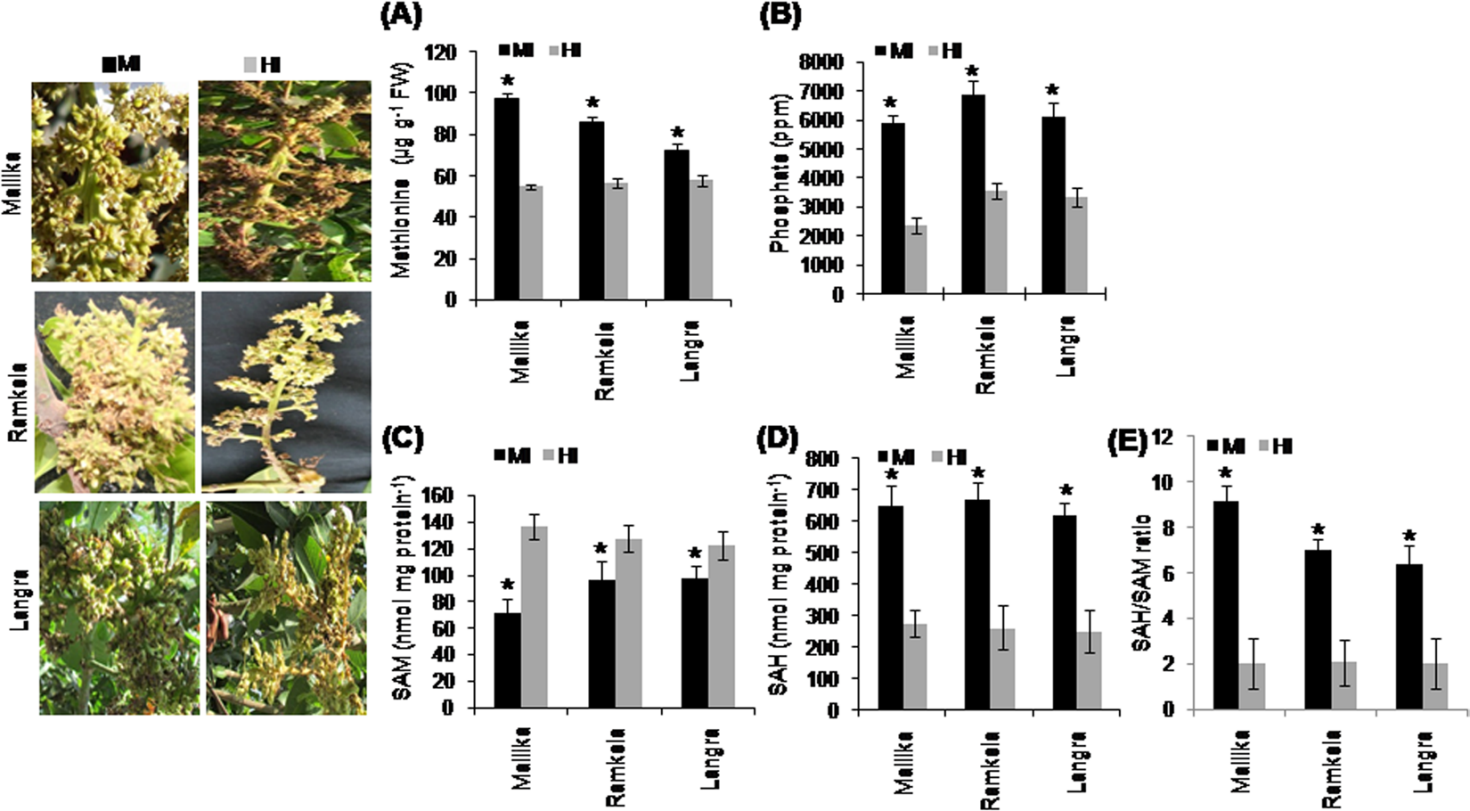
Endogenous methionine, phosphate, S-adenosyl-L-methionine (SAM), S-adenosyl-L-homocysteine (SAH) content and the ratio of SAH/SAM in in mango inflorescence. The malformed and healthy inflorescence of mango cultivars including Mk, Rk and Ln were collected to quantify the endogenous methionine content (µg g^−1^ FW) (**A**) and phosphate level (ppm). (**B**) The endogenous level of S-adenosyl-L-methionine (SAM) (nmol mg protein^−1^) (**C**) the endogenous content of S-adenosyl-L-homocysteine (SAH) (**D**) and S-adenosyl-L-methionine/S-adenosyl-L-homocysteine (SAH/SAM) ratio (**E**) in the malformed (MI) and healthy (HI) floral tissue of mango inflorescence of Mk, Rk and Ln. The MI and HI of Mk, Rk and Ln are shown on the left side of the bar diagrams. Data represent means ± SE of at least 3 replicates. ‘*’Indicates a significant difference between the malformed and healthy inflorescence at the 0.05 level of significance.

The endogenous methionine content of MI was not significantly different from that of HI in any of the cultivars. Moreover, the varietal differences were also not significant (Fig. 2A). Ion chromatography revealed that MI of all the cultivars had significantly higher concentrations of phosphate as compared with their healthy counterparts of mango cultivars Mk, Rk and Ln (Fig. 2B). However, a consistent pattern was not observed in the difference in the concentration of cations (Supplementary Table S1) and anions (Supplementary Table S2) between MI and HI of mango cultivars Mk, Rk, and Ln.

The endogenous levels of SAM were significantly lower in MI as compared with HI (Fig. 2C), whereas SAH levels were higher in MI than HI of all tested cultivars (Fig. 2D). Accordingly, the SAH/SAM ratio of MI (9.13, 6.98 and 6.39) was significantly higher than HI (2.02, 2.06 and 2.05) of cultivars Mk, Rk and Ln, respectively (Fig. 2E).

### Endogenous ascorbate level, dehydroascorbate reductase and the ratio of ASA/DHA

**Figure 3:**
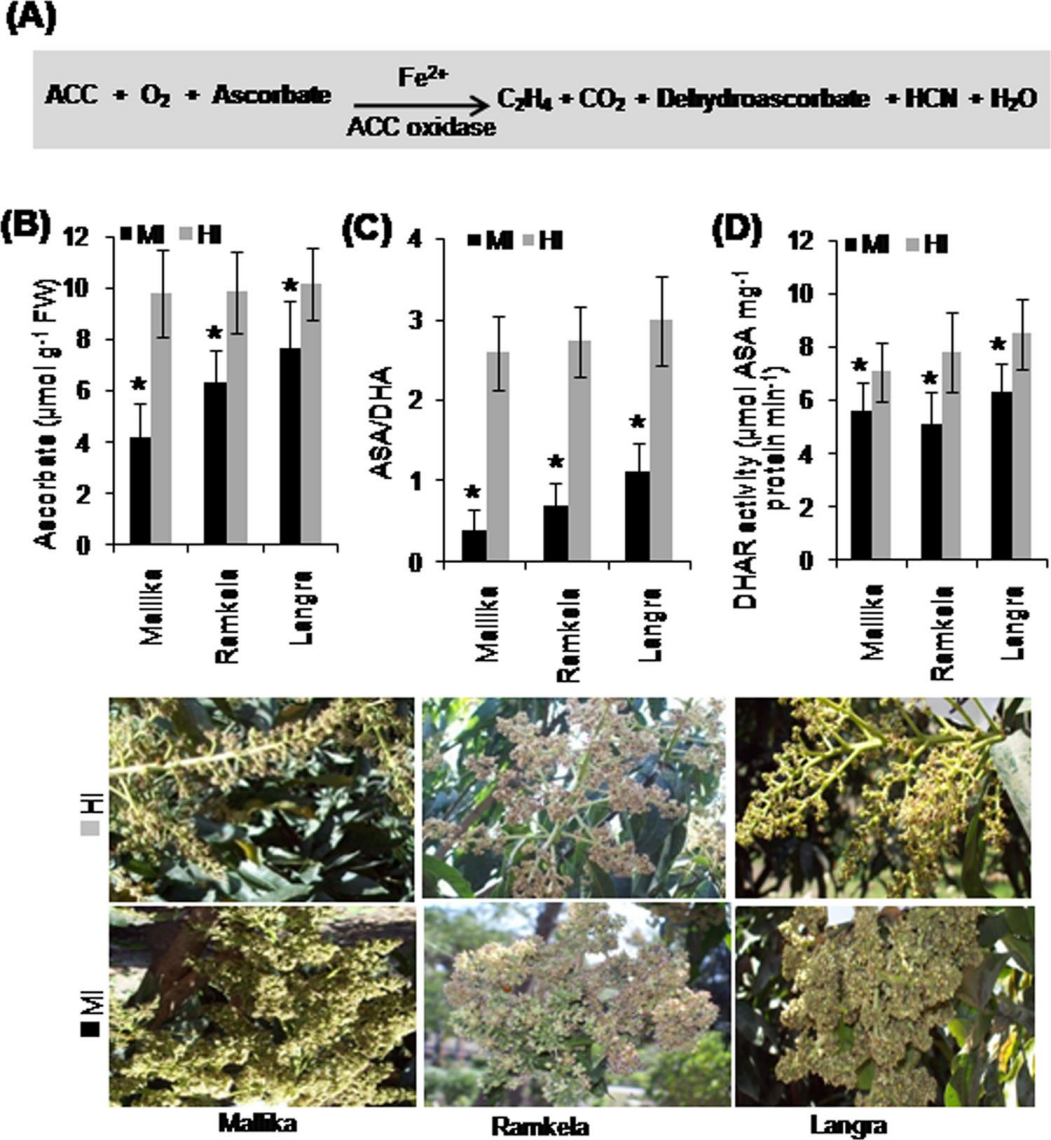
Ascorbate level, dehydroascorbate reductase and ASA/DHA ratio in mango inflorescence. The conversion of 1-aminocyclopropane-1-carboxylic acid (ACC) to ethylene catalyzed by ACC oxidase (ACO) requiring Fe^2+^ and ascorbate as cofactor/cosubstrate. (**A**) The endogenous levels of ascorbate (ASA) (µmole g^−1^ FW) (**B**) the corresponding ascorbate /dehydroascorbate acid (ASA/DHA) ratio (**C**) and the activity (µmole ASA mg^−1^ protein min^−1^) of dehydroascorbate reductase (DHAR) enzyme (**D**) in the malformed (MI) and healthy inflorescence (HI) of mango cultivars Mk, Rk and Ln. The MI and HI of Mk, Rk and Ln are shown underneath the bar diagrams. Data represent means ± SE of at least 3 replicates. ‘*’Indicates a significant difference between the malformed and healthy inflorescence at the 0.05 level of significance.

The final reaction of the pathway that results in the production of ethylene from 1-aminocyclopropane-1-carboxylic acid (ACC) molecule is catalyzed by the enzyme ACC oxidase, which requires Fe^2+^, ascorbate and O_2_ (Fig. 3A). The MI of Mk, Rk and Ln showed significantly lower ascorbate (ASA) (4.16, 6.34 and 7.68 µmole g^−1^ FW) than HI (9.78, 9.83 and 10.18 µmole g^−1^ FW) (Fig. 3B). Moreover, the corresponding ascorbate/dehydroascorbate acid (ASA/DHA) ratio ranged between 0.4–1.1 in MI, whereas it was between 2.58–2.98 in HI of all three mango cultivars (Fig. 3C), respectively. The MI of the three cultivars showed a significant decrease in the activity of dehydroascorbate reductase (DHAR) (5.6, 5.1 and 6.3 µmole ASA mg^−1^ protein min^−1^) as compared with HI (7.1, 7.8 and 8.5 µmole ASA mg^−1^ protein min^−1^) of the mango cultivars (Fig. 3D).

### Endogenous glutathione content and glutathione reductase activity

**Figure 4:**
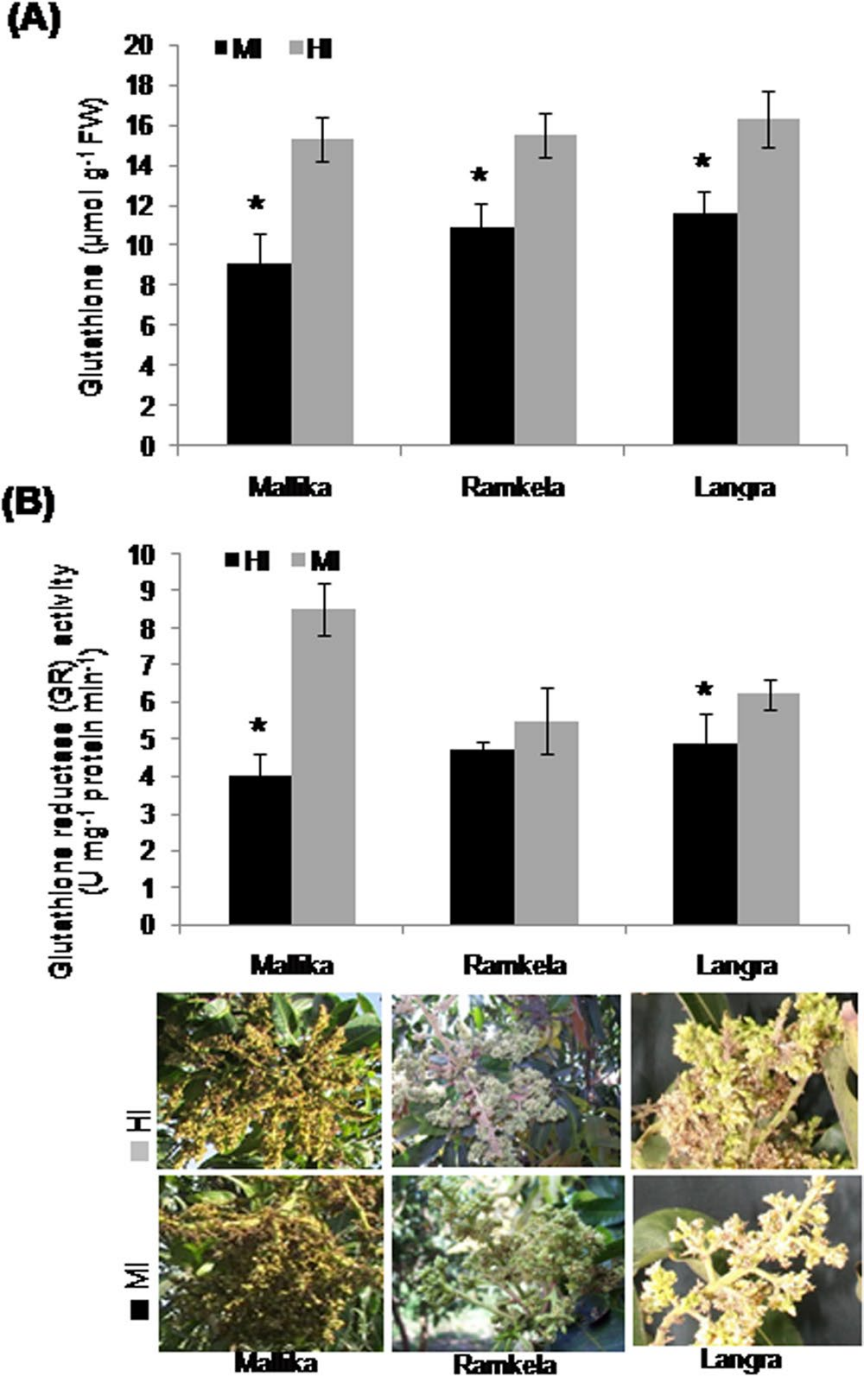
Endogenous content of glutathione and the activity of glutathione reductase in mango inflorescence. The endogenous level of reduced glutathione (GSH) (µmol g^−1^ FW) (**A**) and the activity (U mg^−1^ protein min^−1^) of glutathione reductase (GR) (**B**) in the malformed (MI) and healthy inflorescence (HI) of mango cultivars Mk, Rk and Ln. Data represents mean ± SE of at least 3 replicates. ‘*’Indicates that the difference between the malformed (MI) and healthy inflorescence (HI) was significant at the 0.05 level of significance. The MI and HI of Mk, Rk and Ln are shown underneath the bar diagrams. Data represent means ± SE of at least 3 replicates. ‘*’Indicates a significant difference between the malformed and healthy inflorescence at the 0.05 level of significance.

Reduced glutathione (GSH) levels in the MI of Mk, Rk and Ln, were respectively 9.06, 10.94 and 11.58 µmol g^−1^ FW, which was significantly lower than their HI levels of 15.28, 15.53, and 16.28 µmol g^−1^ FW, respectively (Fig. 4A). Glutathione reductase (GR) activity was also comparatively lower in MI (4, 4.75 and 4.9 U mg^−1^ protein min^−1^) as compared with HI (8.54, 5.5 and 6.23 U mg^−1^ protein min^−1^) in all the three mango cultivars (Fig. 4B).

### Endogenous β-CAS transcript level, β-CAS activity, and unmetabolized cyanide

**Figure 5:**
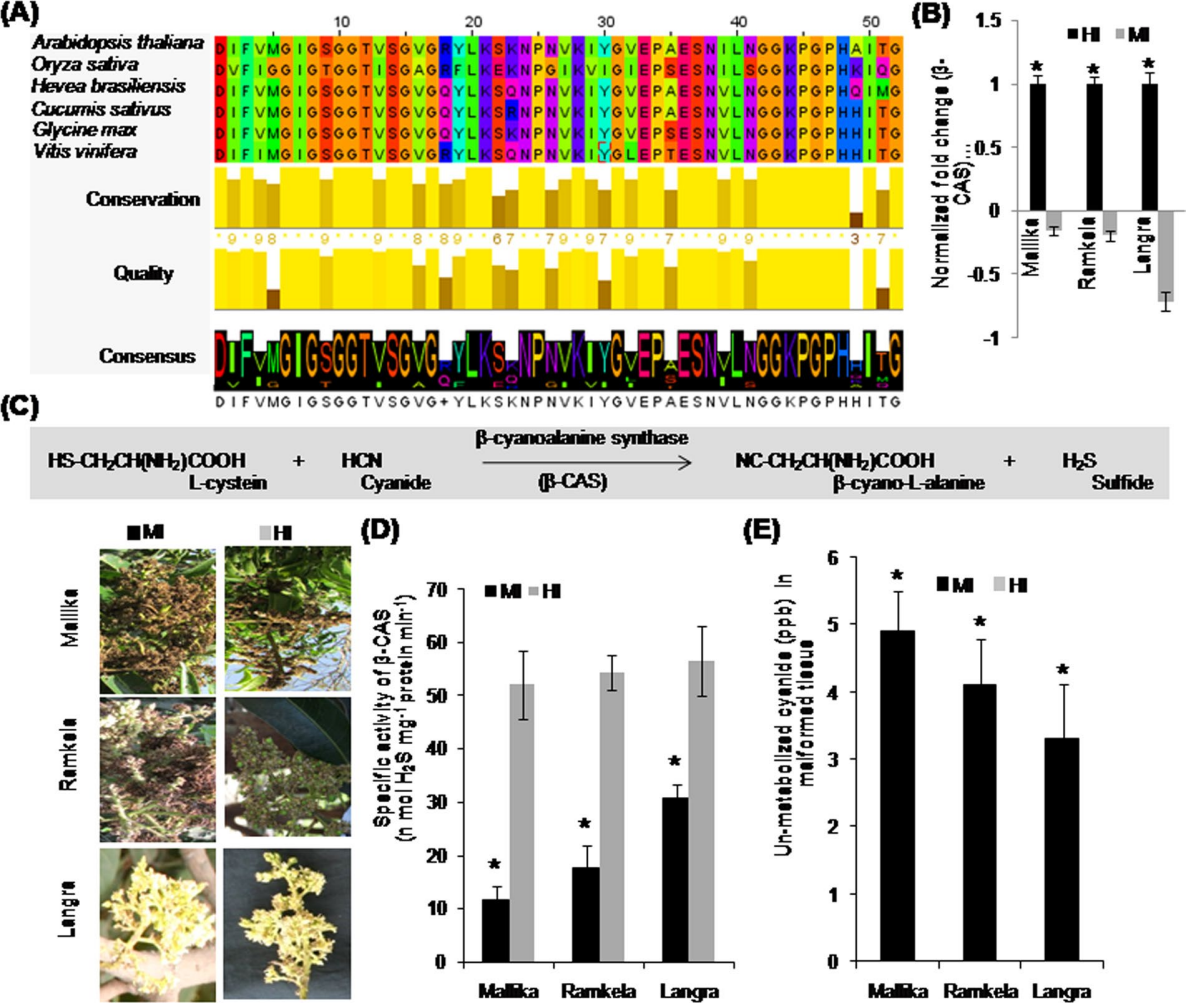
Amino acid sequence alignment of *β-cyanoalanine synthase* gene of various plant species, and the endogenous *β-cyanoalanine synthase* transcript level, β-cyanoalanine synthase activity, un-metabolized cyanide content and succinate dehydrogenase activity in mango inflorescence. The multiple alignment of amino acid sequence of *β-cyanoalanine synthase* (*β-CAS*) gene of plant species (The amino acid sequence of β-cyanoalanine synthase is not available yet in mango genome sequence). (**A**) The *β-CAS* transcripts level in the malformed (MI) and healthy inflorescence (HI) of mango plants of tested cultivars. (**B**) The β-CAS enzyme catalyzes cyanide (HCN) detoxification by utilizing L- cystein, which gives rise to β-cyano-L-alanine. (**C**) The endogenous β-CAS enzyme activity (nmol H_2_S mg^−1^ protein min^−1^ (**D**) in the malformed (MI) and healthy inflorescence (HI) of mango cultivars Mk, Rk and Ln. The endogenous un-metabolized cyanide (HCN) level (ppb) (**E**) in the MI of mango cultivars, however cyanide content in HI was at undetectable levels. The MI and HI of Mk, Rk and Ln are shown on the left side of the bar diagrams. Data represents mean ± SE of at least 3 replicates. ‘*’Indicates a significant difference between the malformed and healthy inflorescence at the 0.05 level of significance.

The multiple alignments of the amino acid sequence of the β-CAS of various plant species revealed conserved regions of 10–48 amino acids (Fig. 5A). The *β-CAS* transcripts in the MI of Mk, Rk and Ln cultivars were 0.71-, 0.19-, and 0.156-fold down*-*regulated as compared with similar observation in HI (Fig. 5B). The β-CAS enzyme, which scavenges potentially toxic cyanide (HCN) in plants by utilizing L- cysteine and giving rise to β-cyano-L-alanine (Fig. 5C), was significantly lower in MI than HI in the mango cultivars (Fig. 5D). ACC is oxidized to ethylene with the release of a stoichiometrically equivalent amount of hydrogen cyanide (Fig. 5E). We observed 4.9, 4.1 and 3.3 ppb un-metabolized HCN in the MI of Mk, Rk and Ln respectively, but we did not detect HCN in HI (Fig. 5E).

### Transmission electron microscopy shows ultrastructure of gum resin ducts and cellular organelles

**Figure 6:**
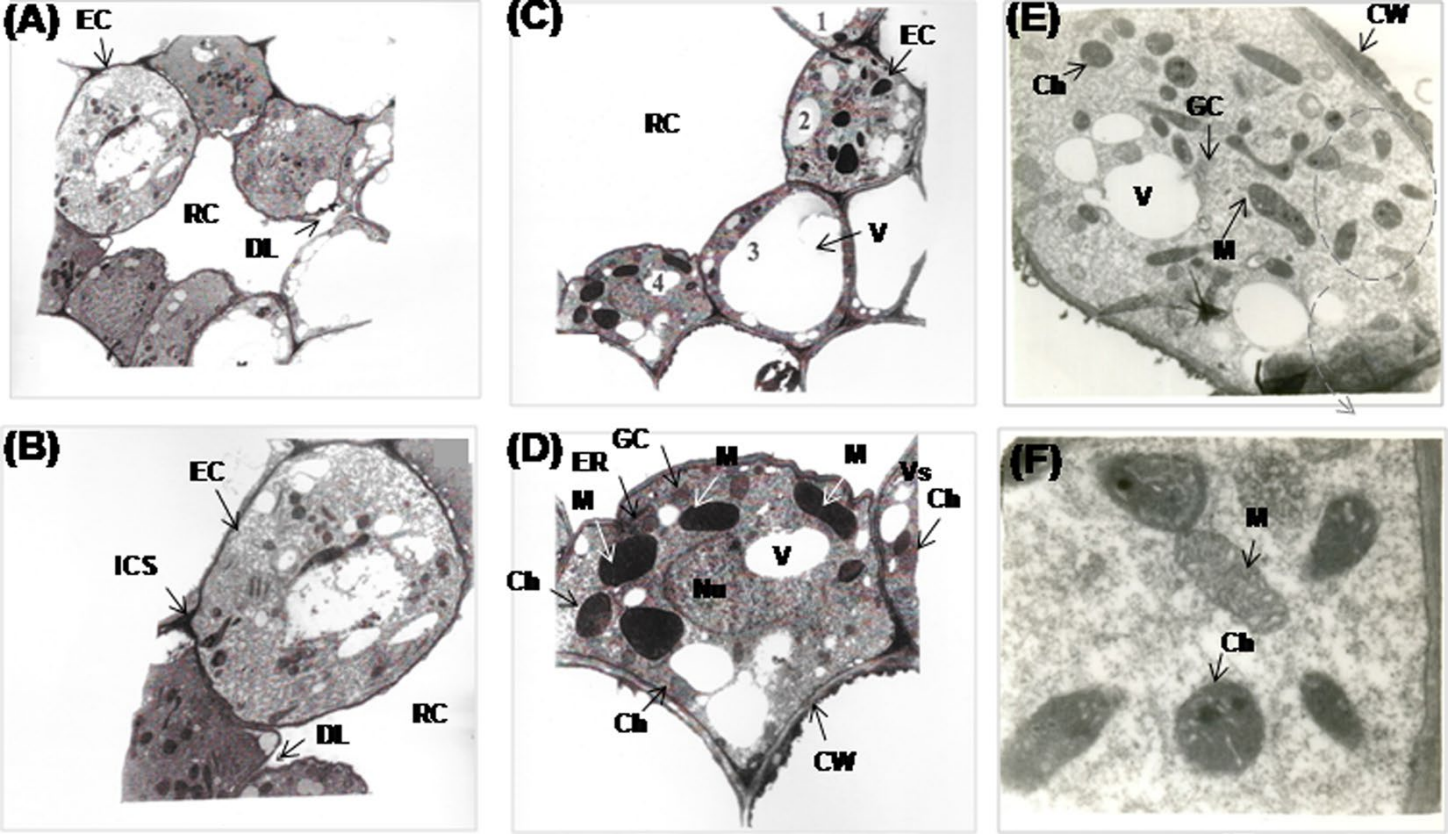
Transmission electron micrograph of malformed and healthy floral tissues of mango cultivar Rk. The cross-section of malformed floral tissues shows a completely disintegrated central cell at the stage of formation of resin canal. The cell-wall regions of the central cell are completely dissolved forming the duct lumen (X 1150). (**A**) Higher magnification view of a cell showed a connection between duct lumen and intercellular space (X 1950). (**B**) The cross-section of malformed floral tissues shows epithelial cells at various stages of differentiation. Cells possess dark cytoplasm and intact cell organelles while mitochondria appear black in the section (X1150). (**C**) Higher magnification view of malformed cell in which the large number of vacuoles are visible in the highly osmiophilic cytoplasm and the mitochondria appears black and irregularly shaped. (X1950). (**D**) The cross-section of healthy floral tissues showing healthy cell organelles (X1950) (**E**) and enlarged view revealing normal mitochondria and chloroplast. (**F**) RC, resin canal; EC, epithelial cell; DL, duct lumen; ICS, intercellular space; CW, cell wall; Ch, chloroplast; M, mitochondrion; Nu, nucleolus; V, vacuole; Vs, small vesicle; GC Golgi complex.

We observed excessive gum-resinosis in the malformed floral tissues (MI) of the mango cultivar Rk as compared with healthy floral tissues (HI); therefore, we used TEM to identify various stages in the development of gum resin ducts in both MI and HI tissues. In the malformed sections, at the initiation stage, the central cell (Fig. 6A) completely disintegrated and dissolved to form a connection between the duct lumen and intercellular spaces surrounding the duct lumen (Fig. 6B) compared with the healthy cells (Fig. 6E). The differentiated cytoplasm of the epithelial cells in MI was dense and dark-stained compared with their healthy counterparts. The cells surrounding the lumen enlarged to form corner lysigenous spaces in MI. The endoplasmic reticulum (ER) in the TEM sections of healthy floral tissues was mostly rough with a continuous array of ribosomes and polysomes. RER profiles were concentrated near the inner tangential wall (Fig. 6B). The epithelial cells surrounding the lumen showed dark cytoplasm and black mitochondria in MI (Fig. 6C,D) compared with HI, which showed normal cytoplasm and healthy cellular organelles, for example, mitochondria and chloroplast (Fig. 6E,F). In the MI, the epithelial cells lining the gum resin canal were thicker and disorganized, and the vacuoles showed an increase in size and number. The mitochondria and chloroplast of the malformed tissues were deformed and blackened, and their number was significantly lower in MI than HI. We also observed a significantly higher number of ribosomes in MI (Table 1).

**Table 1:** Transmission electron microscopy of malformed (MI) and healthy (HI) floral tissue of mango cultivar Rk.

Characteristic	Transmission electron microscopy (TEM)	
	HI	MI
Cell wall	thin	thick
Central vacuole	normal	Enlarged, number increased
Mitochondria	Size is variable both small and large	Disorganized, blackened and their number decreased
Chloroplast	normal	disrupted
Ribosomes	normal	Number increases
Epithelial cells lining the gum resin canal	normal	Thicker, disorganized and blackened

### Dose-response of ethrel and ethylene inhibitors on the incidence of inflorescence malformation

The foliar application of ethrel (0.0, 50, 100 and 200 ppm) on inflorescences of mango cultivars during 2009 and 2010 showed that high ethrel concentrations induced malformed necrotic cells in mango inflorescence of Mk, Rk and Ln in both years. The response was maximum at a concentration of 200 ppm of ethrel. Conversely, the response to a specific ethrel concentration and incidence of necrosis was not consistent for the same cultivar in two different flowering seasons (2009 and 2010). Interestingly, the trend of increasing concentration of ethrel- 50, 100 and 200 ppm reflected the increasing per cent of malformed necrotic inflorescence (Table 2).

**Table 2:** Dose response of ethrel in the development on inflorescence necrosis of mango cultivars.

Mango cultivars	Inflorescence necrosis (%)							
	2009				2010			
	E0	E50	E100	E200	E0	E50	E100	E200
	Mean ± SE	Mean ± SE	Mean ± SE	Mean ± SE	Mean ± SE	Mean ± SE	Mean ± SE	Mean ± SE
Mallika (Mk)	34.01 ± 1.22gh	36.00 ± 0.69 fg	51.04 ± 1.66b	52.00 ± 3.33ab	28.00 ± 1.1f	47.00 ± 1.49cd	49.03 ± 1.5bcd	56.00 ± 1.58a
Ramkela (Rk)	31.00 ± 1.02h	41.00 ± 1.62de	49.02 ± 0.67bc	56.01 ± 0.67a	30.03 ± 1.02f	39.01 ± 1.0e	51.04 ± 1.65bc	52.00 ± 1.03ab
Langra (Ln)	35.00 ± 0.67gh	40.00 ± 1.33ef	45.00 ± 1.66cd	50.00 ± 1.09b	39.00 ± 1.33e	45.00 ± 1.67d	50.00 ± 1.48bc	48.00 ± 0.99bcd

Foliar application of different concentrations of ethrel was evaluated in two consecutive years of 2009 and 2010.

The data in the table are mean ± SE. Means followed by different letters within a row are significantly different in *Tukey’s Post hoc HSD test* at *P* < *0*.*05*. The different concentrations of ethrel are E0 = 0.0 ppm (control); E50 = 50 ppm; E100 = 100 ppm; E200 = 200 ppm.

We used two ethylene inhibitors AgNO_3_ and CoCl_2_ to treat mango inflorescences of Mk, Rk, and Ln cultivars (Tables 3 and 4). Increasing the concentration (from 50 to 800 ppm) of AgNO_3_ and CoCl_2_ gradually reduced the percent malformed necrotic inflorescence in all tested cultivars of mango- Mk, Rk, and Ln.

**Table 3:** Dose response of silver nitrate (AgNO_3_) on inflorescence necrosis of mango cultivars.

Silver nitrate (ppm)	Malformed necrotic inflorescence (%)		
	Mallika (Mk)	Ramkela (Rk)	Langra (Ln)
	Mean ± SE	Mean ± SE	Mean ± SE
Control	72.5 ± 1.18a	69.5 ± 1.61ab	69.0 ± 2.68ab
50	68.7 ± 1.98ab	69.0 ± 2.07ab	67.6 ± 1.64ab
75	67.0 ± 1.54b	69.0 ± 2.25ab	65.0 ± 1.37bc
100	55.9 ± 0.56de	57.6 ± 0.34de	60.4 ± 0.39cd
150	49.3 ± 1.71f	52.3 ± 1.30ef	54.2 ± 2.84 ef
200	36.6 ± 0.98g	50.0 ± 1.79f	49.0 ± 1.61f
250	30.5 ± 1.37h	48.5 ± 0.45f	49.2 ± 0.94f
300	19.2 ± 3.61j	40.7 ± 0.81g	39.0 ± 3.80g
400	7.0 ± 2.73k	23.0 ± 2.68ij	24.8 ± 0.8i
500	0 ± 0l	0 ± 0l	9.0 ± 2.87k
600	0 ± 0l	0 ± 0l	0 ± 0l
800	0 ± 0l	0 ± 0l	0 ± 0l

Foliar application of silver nitrate in concentration raging from 50 to 800 ppm was evaluated during flowering season of 2013.

The data in the table are mean ± SE. Means followed by different letters within a row are significantly different in *Tukey’s Post hoc HSD test* at *P* < *0*.*05*.

**Table 4:** Dose response of cobalt chloride (CoCl_2_) on inflorescence necrosis of mango cultivars.

Silver nitrate (ppm)	Malformed necrotic inflorescence (%)		
	Mallika (Mk)	Ramkela (Rk)	Langra (Ln)
	Mean ± SE	Mean ± SE	Mean ± SE
Control	71.2 ± 2.78a	70.4 ± 1.55abc	69.0 ± 1.26abcd
25	70.75 ± 2.41ab	69.8 ± 1.19abcd	68.0 ± 1.27abcd
50	70.0 ± 1.81abc	70.0 ± 2.45abc	68.5 ± 1.50abcd
75	69.4 ± 2.95abcd	68.8 ± 1.48abcd	66.97 ± 1.37abcde
100	70.3 ± 2.19abc	65.0 ± 2.24abcdef	65.0 ± 1.94abcdef
150	65.0 ± 2.53abcdef	66.25 ± 1.16abcdef	63.63 ± 0.28cdef
200	64.0 ± 3.58bcdef	63.0 ± 2.73def	60.13 ± 1.40efg
300	53.0 ± 2.78h	59.0 ± 0.36fgh	55.3 ± 1.07gh
400	43.0 ± 2.68i	60.50 ± 2.69efg	56.0 ± 2.37gh
600	31.0 ± 3.07j	44.0 ± 2.55i	38.65 ± 3.64i
800	20.0 ± 2.86kl	29.8 ± 0.63j	26.0 ± 3.89jk
1000	9.0 ± 1.03n	16.9 ± 0.46lm	12.8 ± 0.88mn

Foliar application of cobalt chloride in concentration raging from 25 to 1000 ppm was evaluated during flowering season of 2013.

The data in the table are mean ± SE. Means followed by different letters within a row are significantly different in *Tukey’s Post hoc HSD test* at *P* < *0*.*05*.

Ethrel induced percentage of malformed necrotic inflorescence at 400 ppm, and the response was the most pronounced in cultivar Mk followed by Rk and Ln. Interestingly, inflorescences sprayed with high concentrations of AgNO_3_ (>400 ppm) did not show any necrosis indicating that specific concentrations of AgNO_3_ were highly effective in controlling the incidence of malformed necrotic cells (Table 3). Similarly, high concentrations (1000 ppm) of CoCl_2_ significantly reduced necrotic inflorescence in mango cultivars (Table 4).

## Discussion

Malformation of mango inflorescences (MMI) threatens mango productivity and causes significant damage to the mango industry^25,36^. Although *Fusarium* species have been implicated in malformation, the etiology of this disease remains obscure and effective control measures have not yet been identified^4,11,25^. Moreover, fungicides and other tested chemicals have not proved effective in controlling the disease^37,38^. Our previous study revealed higher ethylene levels in malformed vegetative and floral mango tissues as compared to the healthy plants^7^. Cyanide causes abnormal development of flowers and deformation of reproductive organs leading to loss of function^7,8,15,25^. *F*. *mangiferae* increases the endogenous pool of cyanide and ethylene probably by contributing towards its synthesis^9^. Moreover, *F*. *mangiferae* may act through malformation inducing principle (MIP) rather than the toxic principle^39^, suggesting that cyanide can be an etiological agent of mango malformation. Here, we discuss a comprehensive role of cyanide derived from ethylene biosynthesis in the development of mango malformation.

ACS is a key enzyme of regulating synthesis of cyanide, a co-product of ethylene. *ACSs*, encoded by a multigene family, is differentially expressed during plant developmental processes and regulated by various environmental and hormonal signals^40^. *ACS* genes have been studied in diverse plant species^41^. The amino acid sequence alignment of ACS of diverse plant species showed conserved regions of 4–44 amino acids (Fig. 1A). *ACS* transcripts were induced in the senescing petals of *Dianthus caryophyllus*^42^, and increased in the flower buds of Japanese pear (*Pyrus pyrifolia* Nakai)^43^. *ACS* transcripts were abundant in both the detached and attached carnation petals^44^. Moreover, the *ACS* transcripts, ACC synthase activity, ethylene and cyanide production in different stages of flower development coincide with flower senescence^45^. Our data showed a relatively higher accumulation of *ACS* transcripts in MI over HI corroborating the increased ACS activity and elevated cyanide and ethylene levels.

Besides cyanide other important byproducts and/or biomolecules of ethylene synthesis might play an important role in mango malformation. The methionine-ACC pathway for ethylene biosynthesis in plants is well-studied^17^ showing that SAM is converted to ACC and MTA, and MTA is sequentially recycled to methionine^46^. Thus, a small quantity of methionine in the tissue can produce a considerable amount of ACC^47^ indicating that additional methionine is not required to synthesize cyanide and ethylene^29^, which might gradually accumulate within the tissue. The comparatively higher level of methionine in MI than HI of mango plants suggest increased rate of cyanide and ethylene production in the malformed tissues under the hypersensitive stress response. Thus, continuous synthesis of cyanide and ethylene liberates considerable inorganic phosphorous (PPi and Pi) in the step catalyzed by SAM synthetase^25^, and excessive Pi causes the formation of shallow roots reducing plant growth and meristematic activity^48^. Similarly, we have found higher phosphorus concentrations in MI than HI suggesting another link to malformation.

SAM, a vital biological methyl donor, participates in the methylation of biomolecules/volatiles and acts as a substrate for the synthesis of cyanide and ethylene, spermidine, spermine and thermospermine, and nicotianamine and biotin^49^. SAM, a methyl donor in the transmethylation reactions, induces S-adenosyl-L-homocysteine (SAH) formation, which in turn metabolizes to methionine via homocysteine in SAM-dependent methylations^50^. Again, we have demonstrated a lower SAM content and higher SAH/SAM ratio in MI than HI.

ASA, a -water-soluble antioxidant, significantly mitigates oxidative damage^51^, and is a cofactor for many enzymes including ACC oxidase^52^, which catalyzes the final step in cyanide and ethylene biosynthetic pathway^53^. Our data showed low levels of ASA in MI indicating an increased rate of cyanide and ethylene production, which consumes ASA from a constant ‘endogenous ASA pool’ in MI. ASA and DHA play a crucial role in redox homeostasis in the cell^54^, and the ratio of ASA/DHA governs the ability of plant cells to remove reactive oxygen species (ROS) to enhance protection from oxidative damage^55^. According to our results, a reduced DHAR activity in MI might cause a lower ASA/DHA ratio and damage cells. ASA and GSH play an essential role in defensive processes against oxidative damage by generated ROS^56^. Recycling of GSH is catalyzed by GR, which reduces GSSG via NADPH^57^. Cyanide and ethylene alter the ascorbic AA-GSH levels in *S*. *lycopersicum*^58^. ASA synthesis and endogenous levels are down-regulated by cyanide and ethylene^59,60^, which in turn affect the GSH levels. Thus, a significantly lower GR activity and reduced GSH content in mango MI in our study can be attributed to the overproduction of cyanide and ethylene.

A ubiquitous supply of cyanide in higher plants is mainly due to ethylene biosynthesis. Cyanide is a co-product of the production of ethylene from ACC^61^, which is metabolized via the β-cyanoalanine pathway. The β-CAS enzyme, ubiquitously found in every plant tissue, catalyzes the reaction between L-cysteine and HCN to yield β-cyanoalanine and hydrogen sulfide (H_2_S)^62^. *β-CAS* genes have now been studied in many plant species including rice^35,63,64^. β-CAS activity was studied in the leaves and seedlings of various plants^32,65^ and was found to increase in ethylene-treated flowers^66^. β-CAS functions in cyanide detoxification in higher plants including rice and soybean^35,67^ suggesting a negative correlation between cyanide accumulation and *β-CAS* expression^68,69^. In our study, the lower *β-CAS* transcript in MI than HI of mango validates the accumulation of additional un-metabolized cyanide. Increased cyanide levels may block electron transport via cytochrome oxidase (complex IV) and prevent ATP formation. Cyanide and ethylene alter various growth and metabolic processes of plants^70^ and cause the development of resin canals and the surrounding epithelial cells^71^. Cyanide treated flower pedicels of *Nicotiana tabacum* affects the surface area and relative volume of cell organelles and their number^72^. Ethrel treatment has been shown to exhibit decreased flower development by increasing cyanide level^73^. Cyanide and ethylene acts as a modulator of the membrane structure of cellular organelles including chloroplast and mitochondria^74^. In the present TEM study, cells of malformed inflorescence showing black dead mitochondria were observed. The interlink between programmed cell death and malformation in mango inflorescence has not been defined, however there is indication that ROS as signaling molecules could realize programmed cell death^75^, which might be mediated via cyanide. Our TEM sections of malformed floral tissues showed various stages in the development of resin ducts. Moreover, epithelial cells showed distorted cell organelles in addition to deformed black mitochondria, which could be due to the toxic and adverse effects of cyanide.

**Figure 7:**
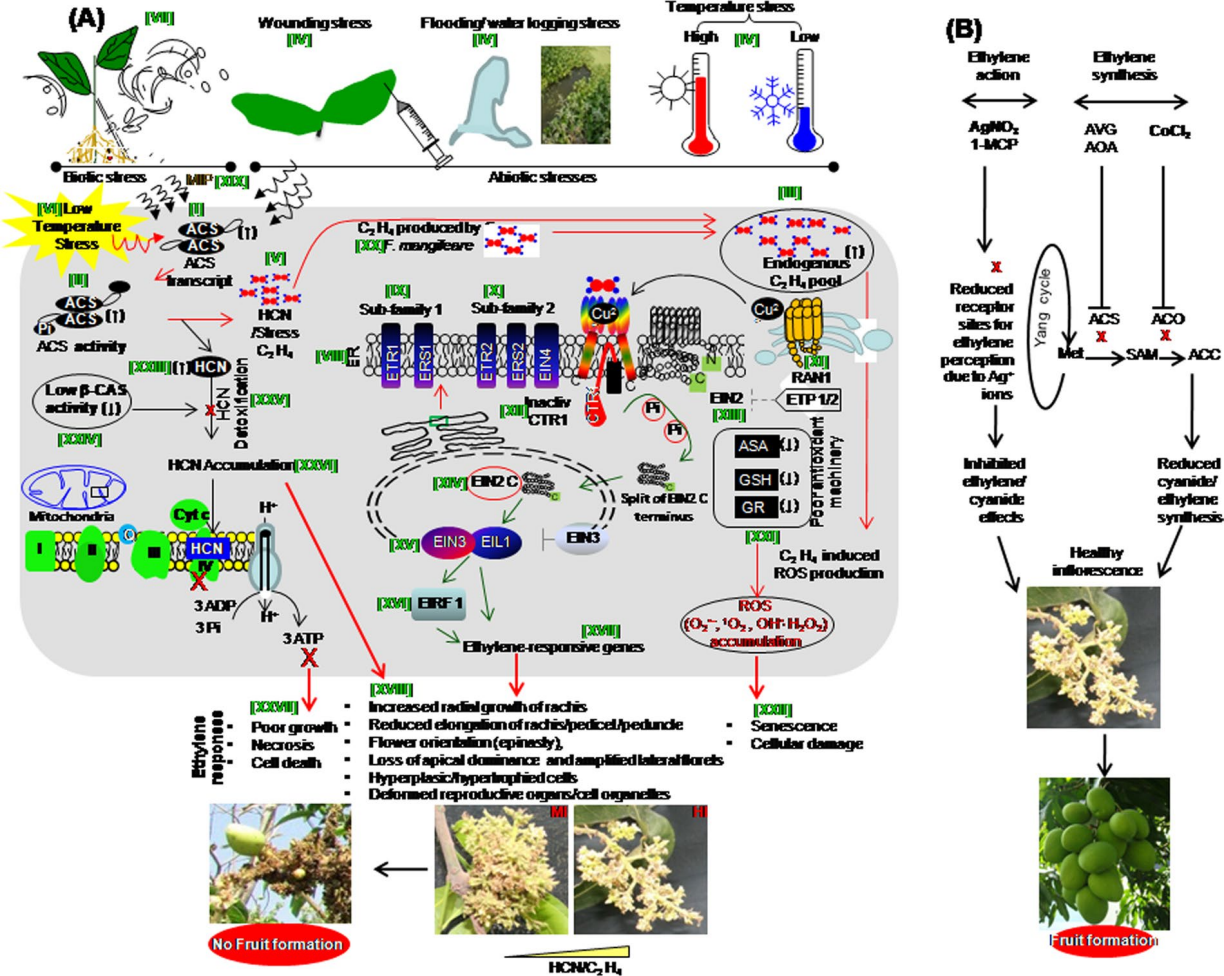
The proposed mechanism for malformation of mango inflorescences (**A**) and its subsequent management. (**B**) The induced *ACS* expression [I] and ACS activity [II] and cyanide and ethylene content [III, XXVI] in response to stress [IV–VIII]. ER [VIII] perceives ethylene by a family of receptors [IX–XI] to inactivate CTR1 [XII]. This leads to dephosphorylation of EIN2 into EIN2 C-terminal [XIII] to induce a transcription cascade of ethylene-responsive genes [XIV] by ERF1 and EIN3/EIL1 [XV–XVII] to show the reponse as malformation symptoms [XVIII]. *F*. *mangiferae* increases cyanide and ethylene pool [XIX–XX, XXVI]. The affected antioxidants system [XXI] accumulates ROS [XXII] causing damaging effects. Cyanide accumulated [XXIII] by low activity of β-CAS [XXIV–XXV] inhibits the complex IV of mitochondrial ETC and ATP formation and causes malformation symptoms [XXVII] (A). Exogenous cobalt chloride (CoCl_2_) and silver nitrate (AgNO_3_) suppresses cyanide/ethylene synthesis and effects to control the disease. (**B**) HI, healthy inflorescence; MI, malformed inflorescence; MS, mango malformation system; MIP, malformation inducing principle; ACS, ACC synthase; β-CAS, β-cyanoalanine synthase; HCN, cyanide; C_2_H_4_, ethylene; ER, Endoplasmic reticulum; ETR1, ethylene response 1; ERS1, ethylene response sensor 1; ETR2, ethylene response 2; ERS2, ethylene response sensor 2; EIN2, EIN3, EIN4, ethylene insensitive 2,3,4; RAN1, responsive-to-antagonist-1; ETP, EIN2 targeting protein; CTR1, constitutive triple response1; EIL1, ethylene insensitive-like protein1; ERF, ethylene response factor; ASA, ascorbate; GSH, reduced glutathione; GR, glutathione reductase; ROS, reactive oxygen species; AgNO_3_, silver nitrate; 1-MCP, 1-methylcyclopropane; AOA, aminooxyacetic acid; AVG, aminoethoxyvinylglycine.

Based on our results and previous studies, we propose a model summarizing the role of cyanide in mango malformation (Fig. 7A), and its subsequent management via inhibiting cyanide and ethylene synthesis and action (Fig. 7B). Multiple abiotic and/or biotic stresses influence the basal cyanide production in the ‘mango malformation system’ (MS)^25,38^ and the ‘system other than mango malformation’ (SOM)^76,77^. Previous studies show that ACS expression is modulated in response to stress in SOM^17,70,78^, which in turn regulates ethylene and thereby cyanide. Ethylene is perceived at the ER receptors to follow the signal transduction cascade of CTR1 inactivation, a downstream transcriptional cascade of EIN2 C, and the activation ethylene responsive genes^79^.

As shown in Fig. 7A, our study on MS shows that MI have induced ACS expression [I] and ACS activity [II] and an elevated endogenous cyanide/ethylene pool [III, XXVI]. Cyanide and ethylene production in response to stress, for example, wounding, waterlogging and very low and high temperatures [IV] is termed as ‘stress induced cyanide/ethylene’ [V]^25^. The stress induced ACS expression corroborates higher levels of cyanide/ethylene in MI under low-temperature stress during winter [VI, XXVI]. Moreover, *Fusarium* invasion as a wounding stress can induce ethylene synthesis in MS [VII]). Endoplasmic reticulum (ER) [VIII] is the main site of ethylene perception by a family of receptors comprising subfamily-I (ethylene response 1, ETR1; ethylene response sensor 1, ERS1) [IX] and subfamily-II (ethylene response 2, ETR2; ethylene response sensor 2, ERS2; ethylene insensitive 4, EIN4) [X]^80^. Moreover, Cu^2+^ (Copper) transporter responsive-to-antagonist-1 (RAN1) deliver the Cu^2+^, which is required ethylene binding to receptor [XI]. Thus, ethylene receptors are inactivated, and the ‘constitutive triple response1 (CTR1) protein kinase’ [XII] causes EIN2 dephosphorylation and consequently its cleavage [XIII]^81^. EIN2 C-terminal section is localized to the nucleus [XIV] in which stabilization/accumulation of EIN3/EIL1 [XV] takes place activating genes downstream of EIN2 inducing transcription cascade of ERF1 [XVI] and ethylene-responsive genes [XVII]^79^ causing the triple response, epinasty, and senescence in mango inflorescence [XVIII]. Earlier, we have shown that low-temperature stress induced cyanide/ethylene production could produce malformation symptoms^7^ [VI] wherein *Fusarium mangiferae* may function through MIP^7^ [XIX]. Moreover, *Fusarium mangiferae* has a potential to produce ethylene itself^9^ [XX], which may add to the ethylene pool in mango inflorescences [III]. The cyanide/ethylene mediate various responses in MS include the loss of apical dominance, increased radial growth of rachis and reduced elongation of rachis and pedicel/peduncle, flower orientation (epinasty), amplified lateral florets, and crowded flowers, which become a thick mass of hyperplasic/hypertrophied cells with deformed reproductive organs^15,25^ and disrupted cell organelles, necrosis and dead cells [XVIII, XXVII]. Stress-induced cyanide/ethylene production involving the generation of ROS^82^ is associated with cellular organelles damage via lipid peroxidation, which requires a functional antioxidant for scavenging the ROS^75^. The present study shows ASA, GSH, and GR are adversely affected [XXI] by stress-stimulated cyanide/ethylene overproduction, leading to a poor antioxidant system, causing senescence, cellular damage and cell death by unscavenged ROS [XXII]. Most plants generate cyanide as a byproduct of the ethylene biosynthesis^64^. Here, in MS the level of endogenous cyanide increases [XXIII] because of the higher pace of ethylene biosynthesis under stress. Moreover, the low activity of β-CAS [XXIV] leads to poor detoxification [XXV] that causes the accumulation of unmetabolized cyanide [XXVI]. Cyanide blocks the complex IV of mitochondrial ETC and inhibits ATP formation^31^, leading to poor growth of MI. The hyper-toxic effect of HCN can be responsible for acute necrosis and cell death of MI [XXVII].

As described in Fig. 7B, cyanide/ethylene effects and synthesis can be inhibited by different inhibitors to protect crop from its various adverse effects on physiological and developmental processes^83^. These inhibitors are useful for biological research to fine-tune the mechanisms involving cyanide/ethylene synthesis and/or signal transduction^60^. The AgNO_3_ and 1-methylcyclopropane (1-MCP) have been identified to inhibit ethylene action and thereby reduced toxic cyanide effects^84^ whereas aminooxyacetic acid (AOA), aminoethoxyvinylglycine (AVG) and cobalt chloride (cobaltous ions) are known to inhibit cyanide and ethylene formation^85^. Silver ion occupies the binding site for copper at ethylene receptors, and 1-MCP blocks ethylene binding of the receptor proteins^86,87^. Silver ion specifically blocks the action of ethylene in its classical physiological responses^88^. Here, our study shows AgNO_3_ reduces incidence of malformation. Cobalt ions promote growth factors alleviate the senescence of aged tissues as it inhibits the activities of ACC oxidase and reduce cyanide and ethylene production^85,89^. Exogenous application of cobalt chloride reduces the endogenous cyanide and ethylene concentration^90^. Our data showed the cobalt chloride application result in reduced incidence of necrotic malformed mango inflorescence. The proposed model will help us to comprehend the physiological and molecular basis of the mechanisms and its subsequent management underlying cyanide coproduct of ethylene as a key player in malformation.

In summary, we propose that cyanide derived from ethylene is a critical agent responsible for the MMI disease. Enzymes that regulate cyanide/ethylene production, and by-products of ethylene biosynthesis in plants, determined in this study, corroborate our hypothesis. Moreover, we have described for the first time, the impact of cyanide and ethylene on cellular morphology in HI and MI. This is the first such study providing molecular-biochemical evidence for the disease. Our data also show that cyanide production with ethylene, and its partial detoxification can be controlled by manipulating the key regulatory enzyme, ACS and β-CAS. This knowledge might be applied to manipulate mango varieties genetically for engineering resistance to malformation.

## Methods

### Plant materials, experimental site, and meteorological conditions

Mk, Rk and Ln cultivars showed different flowering (December to January) and fruit maturity (May to July) time^91^. These cultivars are diversely related each other in a study on nucleotide polymorphisms (SNPs)^92^. The genetic background of these cultivars described earlier showed that Rk cultivar grouped in phylogenetic cluster 5 of structure sub-population I, while Ln and Mk cultivars are grouped in phylogenetic cluster 7 of structure sub-population II^93^. The mango cultivars Mk, Rk and Ln differing in their degree of susceptibility to mango malformation disease were selected for the present study^75^. Of which Mk Rk and Ln are representing highly susceptible, moderately susceptible and less susceptible cultivars^75^. The MI and HI were collected from twenty years old plants of Mk, Rk and Ln cultivars of mango during winter where existing mean temperature varied ‘between’ 10–15 °C, and mean precipitation and relative humidity ranged between 0–0.4 mm and 44–66%, respectively. The temperature during summer ranged from 26–46 °C. The canopy size and trunk dimension of mango cultivars were 10–12 feet and 30–40 cm, respectively. The phenological stages are divided into the growth stages for bud, leaf and shoot development, inflorescence emergence, flowering, fruit development and fruit maturity. All plants used for sampling were at the flowering stage. The experiments were carried out in the Orchard at the Department of Plant Physiology, G.B. Pant University of Agriculture and Technology, Pantnagar, which is located at 29°N latitude, 79.3°E longitude and an altitude of 243.8 m over mean sea level. It belongs to Gangetic Plains Region of Uttarakhand state of North India representing the Terai belt – a lowland region in the northwestern part of India, south of the outer foothills of the Himalayas, which is characterized by tall grasslands, swamps, and hot and wet summers followed by cold winter.

### Alignment of ACS and β-CAS protein sequences

Protein sequence data for *ACC synthase* (*ACS*) and *β-cyanoalanine synthase* (*β-CAS*) genes from different plant species were achieved by BLAST-P searches of the National Center for Biotechnology Information (NCBI) Entrez databases^94^, which are listed in Supplementary Table S3. Multiple amino acid sequence alignments were made using Clustal X software to study the transcript level of *ACS*, and *β-CAS* genes in mango inflorescence^95^ and the primers were designed (Supplementary Table S4) from highly conserved regions for qRTPCR. However, primers for Actin were designed from the CDS sequence available in NCBI database (Accession number: At3G46520).

### RNA isolation and quantitative real-time PCR (qRT-PCR)

Total RNA from mango MI and HI was isolated using TRIzolH Reagent (Invitrogen, http://www.invitrogen.com) as per manufacturer’s instructions, and poly(A)-RNA was isolated^96^. It was used to prepare cDNA using the RevertAid H minus first-strand cDNA synthesis kit (Fermentas, http://www.thermoscientificbio.c om/fermentas) as described previously^97^. The primers for all the defined genes were designed via PRIMER EXPRESS (PE Applied Biosystems, USA) with default parameters. Primers were validated using BLAST tool of NCBI. The dissociation curve was examined after the PCR reaction to check the specificity. A list of primers for qRT-PCR is shown in (Supplementary Table S4). The reaction for qRT-PCR was set up as described earlier^98^ in ABI 7500 Real-Time PCR System (Applied Biosystems, USA). The relative levels of the transcript accumulated for all the selected genes were normalized using α-tubulin like the endogenous control using 2^−∆∆Ct^ method^99^. Biological and technical replicates of each given sample were studied for the analysis.

### Determination of ACS activity and quantification of ethylene and cyanide

The floral tissues (0.2 g) were ground using mortar and pestle in 3 mL of extraction buffer (0.1 M EPPS-KOH buffer, pH 8.5, 10 mM 2-mercaptoethanol, and 10 µM pyridoxal phosphate) at 2 °C. ACS (EC 4.4.1.14) was extracted by the methods of Kato *et al*.^100^ with slight modification. ACC formed in the reaction was assayed by the method of Lizada and Yang^101^. Protein was estimated by using the method of Bradford^102^. Enzyme activity was expressed as the amount of ACC (nmol) produced per mg protein per hour. Ethylene content in the tissues of MI and HI of mango cultivars was quantified by following the method of Nakatsuka *et al*.^103^ using the same set of experimental conditions and gas chromatography as described by Ansari *et al*.^7^. Mango floral tissue un-metabolized cyanide (HCN) was estimated colorimetrically by the method of Lambert^104^ with minor modifications. The vacuum extraction was done for extracting intercellular HCN gas from 2.0 g of MI and HI tissues. The collection flask contained 200 μL of 0.1N NaOH to trap the extracted HCN gas. To 200 μL of trap solution (0.1N NaOH) were added 100 μL 1 M acetic acid, 1 mL 0.25% succinimide/0.025% N-chlorosuccinimide reagent, and 200 μL 3% barbituric acid in 30% pyridine. The reaction mixtures were shaken vigorously for 10 minutes. The absorbance was read at 580 nm, and the results were expressed in ppb.

### Determination of the methionine content, total cations and anions concentration, and SAM and SAH

Floral tissues of MI and HI (0.5 g) were weighed into a 50 mL conical flask containing 6 mL of 2 N HCl and then were autoclaved at 15 lb pressure for 1 hr. A pinch of activated charcoal was added to the hydrolysate. It was boiled and washed with hot distilled water. The filtrate was neutralized with 10N NaOH to pH 6.5. The volume was made to 50 mL with distilled water after cooling ambient temperature and 25 mL of it was transferred to 100 mL conical flask. Subsequently, 3 mL of 10% NaOH and 15 mL of 10% sodium niterprusside (10%) were added. After standing for 10 minutes with repeated shaking, 1 mL of glycine (3%) solution was added. The mixtures were allowed to stand for another 10 minutes with frequent shaking, 2 mL of orthophosphoric acid was added and shook vigorously. Intensity of red color after 10 minutes was read at 520 nm against a blank prepared in the same way without niteroprusside. The methionine content was calculated using standard curve as described by Horn *et al*.^105^ and was expressed as µg g^−1^ FW.

The total cations and anions concentration was determined by ion chromatography. For the analysis of cations, dry floral tissues (0.5 g) mango MI and HI were kept in 5 mL triacid mixture (nitric acid: sulphuric acid: perchloric acid, 1:1:4, v/v/v) for 20 min. The samples were digested using a hot plate in a digestion hood. The dry digested samples were reconstituted in 5 mL of 6 N HCl, and the volume was made up to 20 mL using double distilled water. The samples were then filtered for analysis using ion chromatography using a sample size of 1 mL, Loop size – 100 μL, Metrosep Cation 1–2 column, the flow rate of 1.0 mL/min. The eluent used was 4 mM Tartaric acid plus 0.75 mM dipicolinic acid plus 10% acetone, and the detector was set to full scale to 20 μS\cm.

Anion analysis was carried out by homogenizing dry 0.3 g tissues of MI and HI in 20 mL deionized distilled water. The samples were filtered before analysis. The prepared samples were injected into an ion chromatograph for ion analysis. Analysis of phosphate (${{\rm{PO}}}_{4}^{-3}$) was carried out using a sample size 1 mL for injection, loop size 20 μL, column IC Sep AN2 and IC Sep ANSC, flow rate 1.2 mL/min, eluent 1 mM Sodium carbonate +3.5 mM Sodium bicarbonate +10% acetone, detector setting and full scale: 20.0 μS/cm^106^.

For the estimation of SAM and SAH, floral tissues (approximately 500 mg) of MI and HI were harvested from selected cultivars and instantly stored in liquid nitrogen at −80 °C. The tissue was homogenized in liquid nitrogen and extracted at 4 °C for 20 min with 0.5 mL of 0.1 N HCl. The plant homogenate obtained was centrifuged for 10 min at 4 °C and 16,400 g to eliminate cell debris. SAM (nmol mg protein^−1^) was estimated as described by Van de Poel *et al*.^107^ with slight modification. SAH was determined as described by Bürstenbinder *et al*.^24^ after chloroacetaldehyde derivatization.

### Measurement of ASA and DHA and determination of DHAR activity

Fresh floral tissues (0.5 g) from MI and HI each were crushed and blended in 2 mL of extraction buffer (100 mM, pH 7.0 with 1 mM EDTA) and then centrifuged at 10,000 g for 10 min. The supernatant was collected, and ASA and DHA were extracted and determined as described by Wang *et al*.^108^ (1991) with slight modification. A standard curve in the range of 10–100 nmol of ascorbate was used for calibration. The DHAR activity in the sample was assayed at 265 nm by adopting the method of Nakano and Asada^109^. Protein was estimated^102^ using bovine serum albumin as standard.

### GSH content and GR activity measurements

We homogenized 500 mg tissue each from MI and HI in 2 mL 5% (w:v) sulphosalicylic acid at 4 °C. The homogenate was then centrifuged at 10,000 g for 10 min. After centrifugation, the supernatant (0.5 mL) was added to 0.6 mL of 100 mM (pH 7.0) phosphate buffer, 40 µL of 10 mM 5,5-dithio-bis(2-nitrobenzoic acid) (DTNB), 150 µL of 0.5 mM nicotinamide adenine dinucleotide phosphate (NADPH) and GR (10 units/mL). After 2 minutes at 412 nm wavelength in a UV-vis spectrophotometer, the absorbance was measured to determine total glutathione. GSSG was estimated in presence of 2-vinylpyridine and GSH content was calculated by subtracting GSSG from total glutathione, and was expressed as µ mol g^−1^ FW^110^.

Fresh MI and HI tissues (250 mg) were homogenized in 2 mL of extraction buffer containing 100 mM potassium-phosphate buffer (pH 7.0), 1.0 mM EDTA, 2.0 mM dithiothreitol (DTT), 1.0 mM phenylmethanesulfonyl fluoride (PMSF), 0.05% (v/v) Triton X-100, 10% (v/v) glycerol and 1% (w/v) polyvinylpyrrolidone (PVP) at 4 °C. The extract was then centrifuged at 12,000 g for 20 min at 4 °C. The GR (EC 1.6.4.2) activity was estimated by adding 0.1 mL of crude enzyme extract to 0.9 mL of a reaction medium consisting of 100 mM potassium phosphate buffer (pH 7.5), 1.0 mM ethylenediaminetetraacetic acid (EDTA), 1.0 mM GSSG, and 0.1 mM NADPH 0.5 mM Tris-HCl. GR activity (U mg^−1^ protein min^−1^) was measured as described by Foyer and Halliwell^111^ by monitoring the rate of decline in the absorbance of NADPH at 340 nm (extinction coefficient 6.22 mM^−1^ cm^−1^).

### β-cyano alanine synthase assay and determination of tissue un-metabolized cyanide

One gram tissue from each MI and HI was ground in 0.2 M Tris-Buffer (pH 8.5). The homogenate was centrifuged at 10,000 g for 20 min and the supernatant was collected. The method of Miller and Conn^32^ was followed for β-cyano alanine synthase (EC 4.4.1.9) assay with slight modification. The reaction was performed in a serum capped vials containing 0.2 mL of supernatant containing enzyme extract, 0.8 mL Tris buffer (0.1 M, pH 8.5), 25 mM L-cysteine, and 25 mM NaCN in a final volume of 1 mL. After incubation in water bath at 35 °C for 30 minutes, the reaction was stopped by injecting 100 µL 20 mM *N*,*N*-dimethyl-*p*-phenylenediamine in 7.2 N HCI and 100 µL 30 mM ferric chloride (FeCl_3_) in 1.2 N HC1 through the serum cap. The color developed due to the presence of H_2_S was measured at 650 nm, using sodium sulfide (Na_2_S) as the standard. β-cyano alanine synthase activity was expressed as nmol H_2_S mg^−1^ protein min^−1^. Protein was estimated by the method of Lowry *et al*.^112^. Mango floral tissue un-metabolized HCN was estimated colorimetrically by the method of Lambert^104^ with minor modifications as shown above.

### Transmission electron microscopic study of mango inflorescence

Floral tissues from MI and HI of mango cultivars were fixed in a solution containing 2.5% (w/v) glutaraldehyde and 2.5% (w/v) formaldehyde in 0.1 M NaPO_4_ buffer (pH 7.3 ± 0.05). Ultrathin microtome sections (50–60 nm) were examined using a Jeol 1010 electron microscope (Tokyo, Japan) equipped with a CCD Megaview camera, operated at 100 kV by as described by Shibuya *et al*.^113^. Before TEM observation, these sections were treated with 2% aqueous uranyl acetate for 20 min and lead citrate for 5 min^114^.

### Field trial study to evaluate the response of ethrel and ethylene inhibitors on incidence mango malformation

The MI and HI of twenty years old plants of six mango cultivars Mk, Rk and Ln were selected for conducting a field trial study of ethrel and ethylene inhibitors at G.B. Pant University of Agriculture and Technology, Pantnagar, Uttarakhand, India. The study was conducted during the flowering season in two consecutive years. Ethrel (0, 50, 100 and 200 ppm) treatment was given in the last week of December during the flowering season of years 2009 and 2010. Ethylene inhibitors, such as silver nitrate (AgNO_3_) (50–800 ppm) and cobalt chloride (CoCl_2_) (50–1000 ppm), were sprayed in the last week of December during the flowering season of 2013. Observations were taken in March by randomly selecting approximately a hundred inflorescences. The percentage of MI was recorded from a total of 300 panicles per plant.

### Statistical analysis

One-Way ANOVA unifactorial analysis was performed on each variable. Means were separated by using *Tukey*’*s Post hoc HSD test* to compare significant differences between treatments *P* < *0*.*05*. All statistical analyses were performed with performed with XLSTAT, version 2013.3.05 (Addinsoft Inc).

## Supplementary information

Supplementary information 
