# Supplementary material for: Cyanide produced with ethylene by ACS and its incomplete detoxification by β-CAS in mango inflorescence leads to malformation
Source: Sci Rep. 2019 Dec 4;9:18361. doi: 10.1038/s41598-019-54787-7 (PMC6892883; doi:10.1038/s41598-019-54787-7)
Supplement: Supplementary file 1 — Supplementary information [file 41598_2019_54787_MOESM1_ESM.pdf]

Cyanide produced with ethylene by ACS and its incomplete detoxification by β-CAS in mango inflorescence leads to malformation

Mohammad Wahid Ansari[1,2.4#], Shail Kaushik[2#], Gurdeep Bains[2#], Suresh Tula[1,3], Bhavana Joshi[2], Varsha Rani[2], Ratnum Kaul Wattal[4], Randeep Rakwal[5], Alok Shukla[2], Ramesh Chandra Pant[2**], Renu Tuteja[1] & Narendra Tuteja[1**]

Supplementary Table S1. *Concentration of cations in healthy and malformed tissues of inflorescence of mango (Mangifera indica L.) cultivars. Results are the means of three independent random samples taken from different plants of each cultivar.*

Cultivar	Potassium (ppm)		Mean	Calcium (ppm)		Mean	Magnesium (ppm)		Mean
	Healthy	Malformed		Healthy	Malformed		Healthy	Malformed	
Mallika	10600.00	14966.67	12783.33	7800.00	7550.00	7675.00	3466.66	2066.66	2766.66
Ramkela	12933.33	18200.00	15566.67	4600.00	4766.66	4683.33	3100.00	2600.00	2850.00
Lagra	13966.67	11466.67	12716.67	4566.66	5966.66	5266.66	3866.66	3233.33	3550.00
Amrapali	10700.00	13900.00	11966.67	5400.00	3800.00	4600.00	2433.33	2466.66	2450.00
Bombay Green	13766.67	14600.00	14183.33	4566.66	9000.00	6783.30	2366.66	2833.33	2600.00
**Mean**	12393.33	14626.67	13510.00	5386.66	6216.66	5801.66	3046.66	2650.00	2843.33

CD at 5% (p = 0.05)	Cultivar (C)	Tissue (T)	C x T
Potassium	ns	2007.63	ns
Calcium	2711.84	ns	3835.13
Magnesium	ns	529.71	ns

Supplementary Table S2. *Concentration of anions in healthy and malformed tissues of inflorescence of mango (Mangifera indica L.) cultivars. Results are the means of three independent random samples taken from different plants of each cultivar.*

Cultivar	Chloride (ppm)		Mean	Phosphate (ppm)		Mean	Sulphate (ppm)		Mean
	Healthy	Malformed		Healthy	Malformed		Healthy	Malformed	
Mallika	3360.00	3020.00	3190.00	2793.33	5873.33	6201.66	3056.66	1780.00	2418.33
Ramkela	6786.66	4466.66	5626.66	5563.33	6840.00	5986.66	2233.33	2173.33	2203.33
Lagra	3153.33	4793.33	3973.33	5846.66	6126.66	4333.33	1506.66	1233.33	1370.00
Amrapali	3166.66	2353.33	2760.00	2233.33	2546.66	2390.00	893.33	653.33	773.33
Bombay Green	3620.00	3640.00	3630.00	2086.66	2653.33	2370.00	593.33	620.00	606.66
**Mean**	4017.33	3654.66	3836.00	3704.66	4808.00	4256.33	1656.66	1292.00	1474.33

**CD at 5% (p = 0.05)**	**Cultivar (C)**	**Tissue (T)**	**C x T**
Chloride	1822.07	ns	ns
Phosphate	1122.27	709.78	ns
Sulphate	814.07	514.86	1151.27

Supplementary Table S3. *List of 1-amino cyclopropane-1-carbooxylic acid (ACC) synthase and β-cyano alanine synthase (β-CAS) genes with accession number and amino acid sequences from different plant species grouped as dicotyledonous and monocotyledonous.*

**Species**		**Abbreviation**	**Accession No.**	**Length (A.A.)**	**Taxonomic classification**
ACS	*Arabidopsis thaliana*	*Atha*	AF074928	248	Planta; dicotyledoneae
	Oryza sativa	*Osat*	NP_001053637	483	Planta; monocotyledoneae
	Solanum tuberosum	*Stub*	CAA81748	465	Planta; dicotyledoneae
	Vigna radiate	*Vrad*	AF151961	467	Planta; dicotyledoneae
	Glycine Max	*Gmax*	XP_003550959	517	Planta; dicotyledoneae
	Theobroma cacao	*Tcac*	Eoy15638	468	Planta; dicotyledoneae
	Vitis vinifera	*Vvin*	XP_002269780	469	Planta; dicotyledoneae
	Populus trichocarpa	*Ptricocarpa*	XP_002320015	468	Planta; dicotyledoneae
β-CAS	*Arabidopsis thaliana*	Atha	NP_191703	368	Planta; dicotyledoneae
	Oryza sativa	Osat	NP_001045577	394	Planta; monocotyledoneae
	Hevea brasiliensis	Hbra	AAP41852	366	Planta; dicotyledoneae
	Cucumis sativus	Csat	XP_004138495	375	Planta; dicotyledoneae
	Glycine max	Gmax	XP_003534555	373	Planta; dicotyledoneae
	Vitis vinifera	Vvin	XP_002276013	371	Planta; dicotyledoneae

Supplementary Table S4. *Sequences of primers used for qRT-PCR*

Gene name	Abbreviation	Forward primer sequence	Reverse primer sequence
1-aminocyclopropane-1-carboxylic acid	*ACS*	5′-AGCCTCTCCAAGGACTTC-3′	5′-TGCGTCTGCGACGAGAC-3′
β-cyanoalanine synthase	*β-CAS*	5′-CGAATTGGCAGTGGAGGCACAGTC-3′	5′-AGCATGTGGACCTGGTTTGC -3′
Actin	*ACT*	5` TTGTAGCTCCACCAGAGAGA-3´	5` TCCACATCTGTTGGAAGGTG-3`
